# Pultaceous Exudations and False Membranes

**Published:** 1863-05

**Authors:** 


### PULTACEOUS EXUDATIONS AND FALSE MEMBRANES.

There is perhaps no error which is oftener committed in affections of the throat than that of mistaking the soft, whitish and caseiform exudations, which are observed on the tonsils and pharynx in a variety of diseases, for real false membrane. In scarlatina anginosa, for example, it is only exceptionally that these exudations assume the membraneous form; they are generally much softer, more easily indented, and much less adherent than the pseudo-membraneous patches of true diphtheria.

These distinctive characteristics, on which we have insisted since 1856, are properly noticed by Dr. Hillier, physician to the Hospital for Sick Children, in a recent clinical lecture on Scarlatina, published in the *Medical News and Library*. Describing the condition of the throat in that disease, he says:

"The *throat* exhibits at first a bright red color, with minute papular elevations on the soft palate; the tonsils, uvula, and soft palate usually become swollen, and in scarlatina anginosa, the color is more dusky, and patches of exudation are commonly seen on the tonsils of a dirty white, yellowish or ash color. The patches are soft, and may be readily detached with a blunt instrument; they are much less tenacious and adherent than the false membrane of diphtheria."

In cases of tonsils dependent on gastric disorders, whitish exudations, sometimes of considerable thickenss, but soft and caseiform, are observed. In applying the probang firmly to the parts, considerable portions adhere to it, and not unfrequently the whole mass is brought away. As long as the throat continues inflamed, and the cause of irritation is not corrected, the exudations may, after being removed, continue to reappear. These affections are not unfrequently attended with considerable tumefaction of the sub-maxillary glands; seldom, however, to as great a degree as is observed in diphtheria. In such cases, which are often mistaken for diphtheria, we have found emeto-cathartics sufficient.—*Pacific M. and S. Journal.*

### BROMIDE OF AMMONIUM.

At the late meeting of the British Association for the Advancement of Science, held at Cambridge, a paper was read by Dr. Gibb, "On the Physiological Effects of the Bromide of Ammonium." He said that, "although not complete, his experiments were sufficiently positive in their results to justify him in bringing the subject before the Association."

This salt, he stated, had a tonic, sedative, or anti-spasmodic action, according to the quantity given and the mode of administration; and the structures affected by it were the skin and mucous membrane, and fatty compounds. In producing anæsthesia of the fauces, it was superior to the bromide of potassium, and possessed the power of diminishing fat in the economy, and influencing the arrest of atheromatous changes; and he thought it would ultimately be found of more value for the reduction of corpulency and allied states than any other substance at present known. It has been found to be very useful in some of the milder forms of skin disease, and of equal value with the bromide of potassium as an absorbent in glandular and other enlargements, and superior to it, in some respects, in the treatment of some other forms of disease. Dr. Gibb has also employed it in epilepsy with marked benefit, and also in cases of strumous ophthalmia in the young. Bromide of ammonium is a white prismatic salt, becoming yellow and slightly acid by exposure to air. It is very soluble in water, but only sparingly soluble in alcohol. It is composed of one atom each of bromine and ammonium.—*London Chemist and Circular—Drug. Circ.*

